# Cost-effectiveness analysis of the daily HIV pre-exposure prophylaxis in men who have sex with men in Barcelona

**DOI:** 10.1371/journal.pone.0277571

**Published:** 2023-01-17

**Authors:** Francesc López Seguí, Unai Oyón Lerga, Laura Laguna Marmol, Pep Coll, Angels Andreu, Michael Meulbroek, Guillem López Casasnovas, Oriol Estrada Cuxart, Jordi Ara Rey, Carles Quiñones, Fèlix Pérez, Javier Fernandez, Àngel Rivero, Laura Ricou Ríos, Bonaventura Clotet

**Affiliations:** 1 Hospital Germans Trias i Pujol, Barcelona, Spain; 2 Centre de Recerca en Economia de la Salut, Barcelona, Spain; 3 Fundació Lluita Contra les Infeccions, Barcelona, Spain; 4 Research Group on Innovation, Health Economics and Digital Transformation (IGTP), Barcelona, Spain; 5 AIDS Research Institute-IrsiCaixa, Barcelona, Spain; 6 Projecte dels NOMS-Hispanosida, Barcelona, Spain; Federal University of Mato Grosso do Sul, BRAZIL

## Abstract

**Introduction:**

Pre-Exposure Prophylaxis (PrEP) for HIV prevention has been implemented in several countries. Previous literature has shown that its cost-effectiveness (and, under some specifications, cost-saving character) is dependent on the reduction in price due to generics, the time-horizon and its effectiveness. The intervention has never been studied in Catalonia after the approval of the PrEP, a territory with extensive implementation.

**Methods:**

Economic evaluation of the implementation of HIV pre-exposition prophylaxis using administrative data from Men who have Sex with Men (MSM) who receive the treatment (at the generic price) compared with non-implementation. A deterministic compartmental model and a social perspective with a micro-costing approach over the time horizon 2022–2062 are used. A baseline 86% effectiveness of PrEP is assumed.

**Results:**

Daily oral PrEP is found to be cost-saving: discounted savings in costs are attained after 16 years, and after 40 years they reach 81 million euros. In terms of health indicators, 10,322 additional discounted QALYs are generated by the intervention. Results are sensitive to sexual behavioral patterns among MSM, the price of PrEP (reduced if offered on-demand), its effectiveness and the discount rate.

**Conclusions:**

The use and promotion of PrEP in Catalonia is predicted to result in substantial health and monetary benefits because of reductions in HIV infections. Short-term investments in the promotion of PrEP will result in important cost-savings in the long term.

## Introduction

Human Immunodeficiency Virus (HIV) is an important disease throughout the world. Having caused and still causing large human losses -690,000 deaths as a result of Acquired Immunodeficiency Syndrome (AIDS) in 2019 throughout the world-, and in spite of a significant reduction in new HIV infection diagnoses reported globally, it continues to affect severely populations at risk. A demographic dissection of affected citizens depicts a strong incidence in certain population sectors: notably, Men who have Sex with Men (MSM), who have increased their share in new infections over time. In 2020, 1,925 new HIV infections were reported in Spain, which represents a rate of 4.07/100.000 inhabitants, Transmission in men who have sex with men (MSM) was the most frequent transmission category (55,2%) [1,2].

In this light, the use of the Pre-Exposure Prophylaxis (PrEP) for HIV prevention has been implemented in several countries. PrEP consists of antiretroviral (ARV) medication based on Tenofovir Disoproxil Fumarate and emtricitabine (TDF/FTC), taken by HIV-negative people in order to avoid HIV infection. Eighty-six countries have put in place different programs to introduce it, with large heterogeneity in terms of coverage and target populations. The PrEPWatch initiative, powered by the AIDS Vaccine Advocacy Coalition, gathers up-to-date information on the regime, the degree of utilization, the eligibility criteria, the status of the drug (branded or generic) and the research programs conducted in the aforementioned countries [3]. Close to 5,000 patients were receiving PrEP in Spain in eight Autonomous Communities and one Autonomous City, of which 2,400 were based in Catalonia [4].

Previous literature, with diverse environments of application and using different methodologies, has shown that the cost-effectiveness (and, under some specifications, cost-saving character) of PrEP is largely dependent on the reduction in price due to generics, the time-horizon and its effectiveness. A study performed in the Netherlands finds that PrEP is cost-effective, showing significant differences in the result according to the kind of regime (Incremental Cost-Effectiveness Ratio (ICER): 11,000€ per Quality-adjusted life year (QALY) gained for daily PrEP; 2,000€ for on-demand) [5]. Another study performed in Germany shows that PrEP is cost-saving after 11–15 years (assuming a 85% effectiveness and performing a sensibility analysis for price reduction between 10% and 60% of branded ARV/PrEP drugs) [6]. In the UK, research has shown that 80% of the simulations attained cost-effectiveness over a lifetime horizon [7]. Conducting a prospective study, evidence from France and Canada shows that the yearly costs accrued from avoiding one infection are 75,258€ with no price reduction, 39,970€ with generic TDF/FTC [8]. Also performed in the Netherlands, the most recently reviewed study shows that 92% and 73% of the simulations were cost-effective, of which 52% and 23% were cost-saving (respectively, without and with risk compensation) [9]. In Catalonia, although at the time of the study PrEP was not approved in Spain nor was the generic drug available yet, some research suggested that PrEP would be cost-saving under a certain assumption on price reduction and its annual cost would range between €25.3–46.7 million/year (on demand PrEP), and €42.9–78.7 million/year (daily basis PrEP) [10].

Although all this evidence suggests scenarios in which the intervention can be cost-effective, the case has never been studied in Catalonia, a territory with extensive implementation of PrEP after its approval. In this context, using a deterministic compartmental model, this study aims to provide an economic evaluation of the introduction of daily PrEP for MSM in BCN Checkpoint (a community-based centre for the detection of HIV and other sexually transmitted infections located in Barcelona, thus in an urban setting) alongside the Hospital Universitari Germans Trias i Pujol (HUGTIP), both serving users from all over Catalonia, in which a large share of the total PrEP-treated people is monitored [11].

## Methods

### Model

The epidemiological model used is a deterministic compartmental model inspired by Nichols et al. (2016) and Van de Vijver et al. (2019) [5,6]. Three main groups are defined in the population: people who take PrEP and people who do not, and individuals infected, diagnosed and treated with ARV. Within the first two groups, an individual can either be infected and undiagnosed, or susceptible to be infected. Once an individual gets infected, the disease status is divided in nine infection stages (S1 Fig). Within the group of susceptible people, a subdivision in two groups is done according to the level of sexual activity. Only people in the most sexually active group (more than 10 different sexual partners a year) qualify to be administered PrEP through the public health system, as it is assumed that they do fulfill at least one of the other conditions (S1 Table).

The model is fully identified by a group of differential equations (S2 Fig and S2 Table) which calculate the number of susceptible individuals with and without PrEP, the number of infected, undiagnosed individuals with and without PrEP, and the number of treated individuals. The time horizon is chosen to be 40 years, as this is the remaining life expectancy at the average age of diagnosis of HIV. A baseline 86% effectiveness of PrEP is assumed. Regarding the retention rate, data from the PrEP monitoring System in Spain, is used (91%) [12]. Finally, in order to construct the cost-effectiveness analysis, each compartment is assigned a cost and Quality-Adjusted Life Years (QALYs). These indicators adjust remaining life years to account for morbidities (S3 Table). Costs and QALYs are discounted at a 3% yearly rate at baseline [5]. A counterfactual in which PrEP is not put in place is constructed, and the relevant indicators are constructed from a comparison between both scenarios. This study follows the Consolidated Health Economic Evaluation Reporting Standards (CHEERS) guidelines [13].

### Costs

A micro-costing through a social perspective approach has been adopted. The unit costs and the degree of utilization of each component in annual PrEP and ARV treatments have been retrieved from current treatment data from the hospital pharmacy service of HUGTIP, at the laboratory prices financed by the Catalan Health Service, the organism in charge of contracting health services. The costs of PrEP are borne fully by the National Health System (NHS), which provides them free of charge through the hospital pharmacy services or authorized care centres. The global, itemized annual costs of PrEP and the HIV treatment for an HIV-infected person (drugs administered and the complete medical monitoring provided alongside) are shown in the supporting information (S4–S7 Tables).

For a person receiving PrEP, a first visit to a specialized practitioner (76.60€) and three subsequent visits (to initiate the treatment and for the follow-up, scheduled quarterly) for a global amount of 165.03€, are organized. HIV serologies, alongside blood and urine test, are done quarterly (28.76€ and 493.12€, respectively), and basal and quarterly tests for STI detection (syphilis, gonorrhoea and chlamydia), summing up to 341.52€. Finally, basal hepatitis A and B serologies, as well as a yearly Hepatitis C serology (costs included in the initial visit). Close to one fourth of the yearly costs of PrEP accrue from the TFD/FTC tablets (28€ monthly, at the generic price, for a global amount of 336€). The monitoring of PrEP is done quarterly. All in all, the global annual costs for a typical individual in the first year of a daily PrEP regime in Catalonia add up to 1,433.84€. This quantity reflects the prescribed and expected usage of the public medical services in the absence of any adverse event.

Alternatively, a HIV-infected individual may enter treatment for an average yearly value of 8,534€ during his first year. Subsequent years imply a total cost of 7,451.42€. Two initial visits (one with the specialized practitioner, one with the hospital pharmacy service) are scheduled (153.20€), as well as 4 quarterly visits for a global amount of 220.04€. A confirmatory HIV test and Hepatitis A, B and C Virus, syphilis and toxoplasma serologies are included in the costs of the initial visits. Two initial clinical analysis (first visit clinical test, and STIs clinical test, 238.16€), five subsequent analyses (396€), a CD4/CD8 test per visit (147.50€), a yearly syphilis serology (6.62€), and a basal HIV genotyping (340€), conform the rest of the costs. Eventually, the monthly ARV drugs, which add up to 7,032€ (586€ per month, at the price agreed by the Catalan Health Services, SCS) constitute 82.39% of the global costs.

In order to enrich the analysis and expand the perspective of the economic evaluation, the introduction of social costs into the model is considered.

To compute the costs of work absences and transport costs (of which the later will not be accounted for as the costs of transport per se, but rather as time off work as well), the assumptions on waiting times by Ouellet et al. [14] will be employed: 4 hours per outpatient visit to the medical centre. Consequently, the average number of visits times the hours spent there times the average labor costs in Catalonia times the number of people in the relevant treatment times the employment rate gives the global cost of work absence per period. An important caveat, though: visit counts depend on the year of treatment (first or subsequent). Yearly, for a working individual on PrEP, these costs are 353.72€ for all years, for average hourly labor costs of 22.10€ in Catalonia in 2019, according to the National Institute of Statistics. For a HIV-infected worker, 402€ or 482.40€ in the first year (depending on whether the first visits to the pharmacy and the practitioner are scheduled on the same day), and 176.80€ for the following years. According to the National Institute of Statistics, the global employment rate in Catalonia was 59.37% in 2019.Implementation, awareness, uptake and retention costs are not considered. All data refer to 2019. Ethical approval and consent were not required.

## Results

Under baseline specifications, the implementation of PrEP is found to be cost-saving (and thus, cost-effective) for the relevant time horizon of 40 years. Savings in costs are realized after 166 months (13.83 years) of implementation, 196 months if costs are discounted at a 3% rate yearly (16.33 years). After 40 years of PrEP scale-up, 81,032,564€ are saved globally, out of which 93,782,731€ are saved from the healthcare payer perspective, and 12,750,167€ are additional costs borne by the patients, who incur losses from work absences and transport costs. It is essential to note the duality in cost dynamics: the intervention is cost-saving globally and for the public health system but generates extra social costs as a result of the continuous medical monitoring of PrEP, which implies more outpatient visits than the counterfactual.

**Fig 1:**
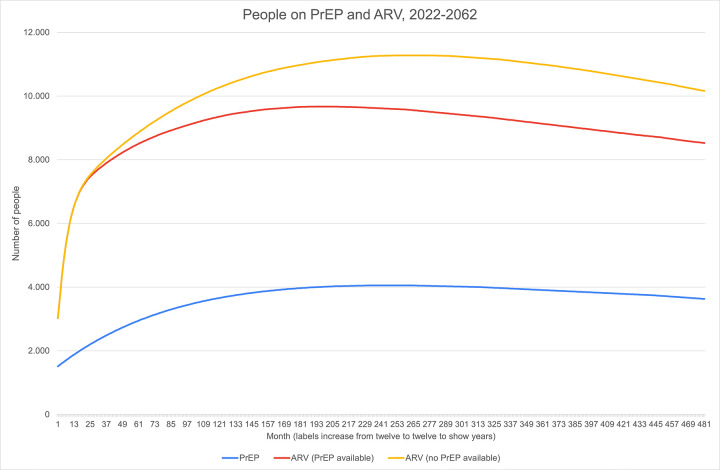
People on PREP and ARV, 2022–2062. Monthly evolution of the number of people on PrEP (blue), and ARV therapy in both scenarios (with PrEP, in red; without PrEP, in yellow).

Furthermore, 23,372 additional QALYs (10,322 discounted annually at 3%) are obtained by using PrEP as a prevention mechanism. No ICER is computed because PrEP generates sizable health benefits at a lower cost than the counterfactual. Fig 1 gathers the evolution of people on PrEP, and ARV therapy in both scenarios, illustrating that introducing PrEP, *ceteris paribus*, implies fewer people on ARV from the third year of implementation because of averted infections.

With respect to costs, ARV drugs for an HIV-infected MSM oscillate over 79–80% of global costs (81–82% of healthcare costs), while PrEP costs oscillate over 1.6% (1.7–1.9% of healthcare costs). It is important to recall the centrality of complementary testing and medical monitoring alongside the daily intake of TDF/FTC, which correspond to a significant share of global costs (for both individuals on PrEP and HIV-infected with antiretroviral therapy) (S3 Fig).

### Sensitivity analysis

**Fig 2:**
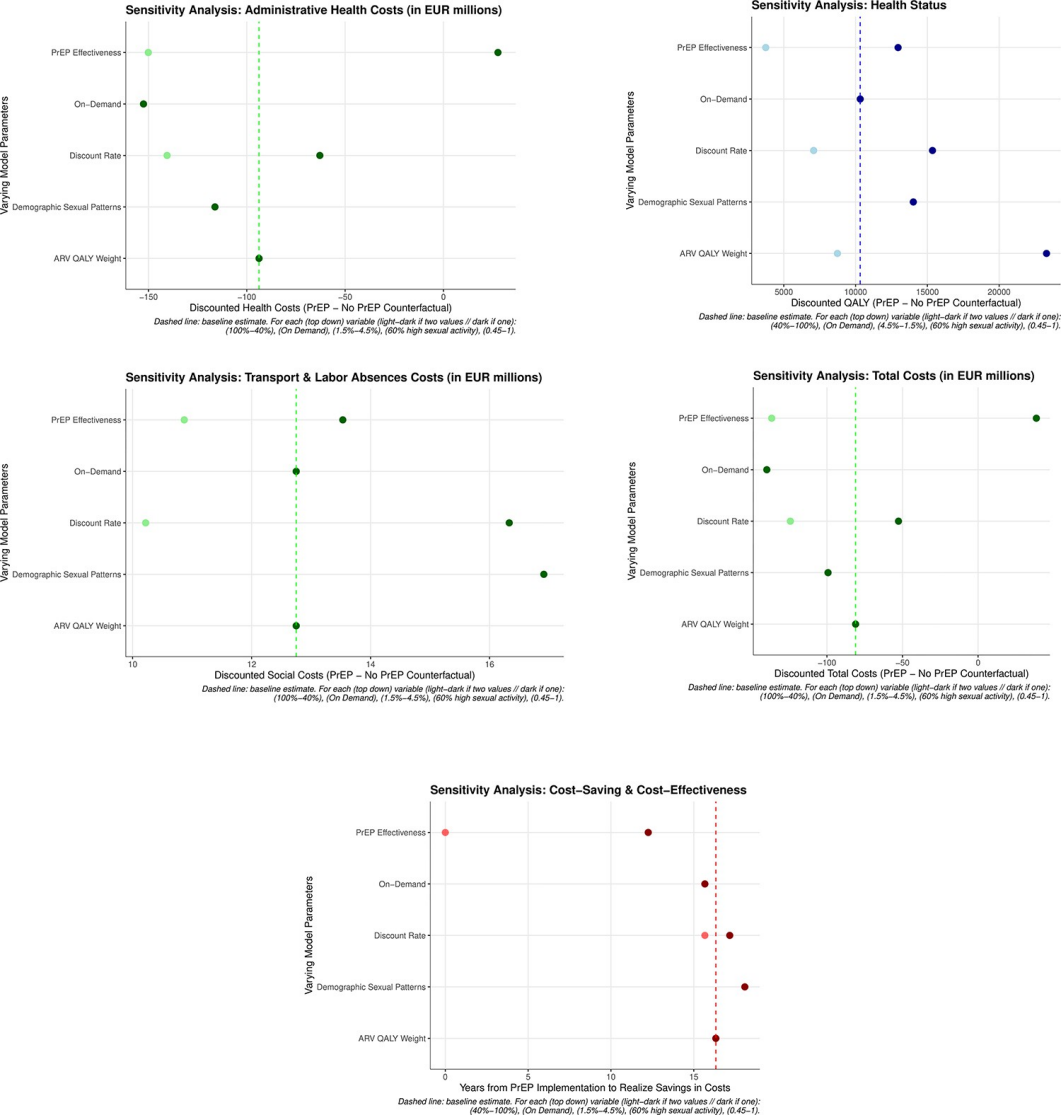
a,b,c,d,e. Administrative health costs, health status, transport and labor absences costs, total costs, cost saving and cost-saving & cost effectiveness.

The conclusions are especially sensitive to the discount rate (range 1.5%-4.5% yearly), which respectively modify discounted additional QALYs and savings in costs by 48.77% and 53.28%, and -31.43% and -35%. In either case, the intervention remains cost-saving for the whole range, between 188 and 206 months of initial implementation (Fig 2).

Changing one or more utility weights for QALYs in each compartment does not generate significant changes in discounted additional QALYs (8,024–11,522; baseline 10,322), except for the case of ARV-treated. Infections averted by PrEP imply systematically lower people on antiretroviral therapy, thus generating substantial health gains as measured by QALYs when the utility weight goes down to 0.45 (23,300, a 126% increase). Cost-savings materialize at the same moment as baseline.

When accounting for different demographic estimations, no significant change is appreciated in terms of QALYs, savings in costs or time to attain these savings. However, a recalibration in terms of sexual patterns strongly impacts health and monetary indicators: 14,020 additional discounted QALYs, 99.2 discounted million euros in total savings (which start in the 18th year), of which 116.2 million euros are saved by the healthcare payer, and 16.9 million of extra costs accrue to society in the form of work absences and transport costs.

Pivoting over the effectiveness of PrEP (40%-98%-100%), against the 86% in baseline, significantly affects savings in costs. Additional discounted QALYs variation with respect to baseline range from -63.77% to 25.55%. Under the unrealistic specification of 40%, PrEP is not cost-saving, and yields an ICER of 10,321€/QALY. On the one hand, given the current structure of PrEP and medical monitoring, such a low effectiveness is not realistic. On the other hand, individual effectiveness for fully-adherent patients is close to 100%, so the complete effectiveness may be feasible for a sufficient degree of compromise in the daily intake, thus saving 136.6 million euros and generating 12,959 QALYs (with respect to the counterfactual, not with the PrEP scenario under baseline specifications). However, the marginal impact from assuming effectiveness to be 100% with respect to the 98% specification is negative in savings: the marginally averted infections (and costs in ARV) do not compensate for the additional costs of keeping everybody in PrEP once they start).

Finally, a reduction in price of 50% following the approval of an on-demand PrEP regime, as assumed in other studies [5,9], does not affect health outcomes, but generates healthcare savings of 152.4 million euros, which start to accrue as soon as 8 years and 8 months after deployment. The results obtained can be extrapolated to an increased-effectiveness scenario (98%), thus resulting in a total cumulative saving of 218 million euros.

## Discussion

The number of people undergoing PrEP in the Catalan population was 2,400 by the time of the study, constituting approximately half of all persons treated in Spain. Applying the same deterministic compartmental mathematical model used by Nichols et al. and Van de Vijver et al., and in line with other studies, favourable cost-effectiveness results have been obtained, that estimate a saving of 93.8 million over a 40-year horizon for a daily regimen and considering, for a conservative scenario, an effectiveness of 86% taken as a reference from other studies [5–9]. Building on the paper by Reyes-Ureña et al [10], our contribution enriches the debate in two main directions. First, it fits the model with actual data on MSM from the Hospital Germans Trias i Pujol, compared to an estimated stock of eligible men before the PrEP was approved in Spain. Besides, we leverage precise micro-costing data for the generic PrEP, which is an essential feature to assess cost-effectiveness, and which had not been developed at the time the aforementioned paper was published.

It is worth highlighting the weight of the direct costs of the drugs on the result (above 70% across the time horizon scanned). Thus, the cost savings are due in part to the start of PrEP in Spain, in November 2019, using the generic presentation of the drugs, which made the global annual cost of PrEP in Spain for a person during the first year of treatment to significantly decrease to €1,433.84. With no adjustment, the difference in PrEP costs between the available literature in other countries and this study varies between €-600.93 (generic drugs and daily administration regimen) [6] and €5,966.16 (daily regimen, brand name drugs) [5,9]. Even when the heterogeneity in PrEP administration is accounted for, the difference in prices is still relevant for the available literature. The underlying reasons can be due to the divergent prices for the drugs, but also on the national guidelines of medical monitoring and clinical testing [15,16].

It should be noted that long-term cost savings will depend on several factors. On the one hand, the evolution of the HIV incidence curve must be analysed. Catalonia has followed a decreasing trend in new diagnoses since 2010, with a reduction in the global incidence rate from 11 to 6.1 cases per 100,000 people in 2019 [17]. Although in our model the infection rate is corrected for the change observed in recent years, if the curve further decreases in the years following the study, the cost-effectiveness results obtained could be altered. Another factor to be considered are the new therapeutic alternatives (new drugs and presentations), which once marketed could affect both the effectiveness of the treatment and its cost.

This model has three main strengths. First of all, it enables the dynamic resolution of the model from the current observational data in the relevant setting, thus making its real-world applicability an essential trait. Then, it permits a long-term, prospective economic evaluation of the impact of PrEP. Accounting for any relevant change in the epidemic, the costs, the treatments or an exogenous shock is easy within this framework, by creating a break in the parameters after the relevant time point. Finally, the administration of PrEP in this model is consistent with the extent to which it has been scaled-up in Catalonia: the national eligibility criteria are respected, and the constraint imposed by limited financial and real resources is shown by a limited take-up rate for eligible people. Furthermore, this methodology is in accordance with relevant contributions in the European literature, as aforementioned, in countries in which the HIV/AIDS epidemic is concentrated among MSM [5,6].

Several limitations are to be identified and tackled in future research. The model does not include stochastic, individual or behavioral features, losing some nuances in sexual patterns and thus transmission patterns. Besides, it does not account for risk-compensation: the introduction of PrEP may encourage sexual behaviours with higher risk by some individuals (lower condom usage rates, for instance), increasing comorbidity through secondary STIs and thus increasing the overall social and healthcare costs of the intervention, although some studies show no significant increase on STI incidence [18]. Finally, the different endogenous changes in infectivity and force of infection as a result of the evolution of the simulation and the count at different stages of the epidemic are not taken into account. Past local data on incidence and prevalence rates slightly diminish this problem. Besides, the geographic scope of the study limits the impact of this problem, since sexual encounters with people who are not included in the count of the model are highly probable: it is not a fully closed sample, so adjusting for the force of infection at different levels of sexual activity does not imply a significant departure from baseline. Nonetheless, these concerns do not imply a significant bias or inconsistency in the results. Secondary bacterial STIs are cheaply treated with generic antibiotics, so no significant budget effect should be expected. Nevertheless, it should be taken into account that the 40-year projection of the study may face the resistance of several pathogens as a barrier, which may become expensive in the future. Infection with Hepatitis C virus could be more problematic because of the cost of the treatment, but yearly serologies included in the PrEP regime may limit the impact of its transmission.

Eventually, another caveat may jeopardize the analytical effort presented here: the distortions created by the COVID-19 in terms of mortality, reduced medical coverage and diagnoses [19,20] and reduced sexual interactions because of the lockdown in Catalonia. This problem could be tackled by accounting for these shocks in terms of the calibration of the parameters of the model, but since this simulation starts prospectively from 2022, when the vaccination coverage in Spain will be significant and the stringency of measures very limited, no modification will be implemented. Also, there is no evidence of a correlation between incidence in SARS-CoV-19 and HIV, although some references indicate that the infection with the aforesaid coronavirus may result in more severe symptomatology for HIV-infected people [21].

Frictions in these costs arising as a consequence of such contingencies were not considered in the analysis, as they are largely dependent on behavioral unobservable and unpredictable events. Besides, if more granular microdata were readily available, transport costs could be associated with direct costs internal to the patients, as well as with traffic and environmental externalities for society. HUGTIP is not an urban hospital, so these costs could be important to the analysis. Further research could expand the ideas hereby presented.

## Conclusions

Daily oral HIV pre-exposure prophylaxis targeted at-risk MSM at BCN Checkpoint and the Hospital Universitari Germans Trias i Pujol currently covers around 3,000 people. Under its current specifications in Spain and Catalonia, this study has provided a cost-effectiveness evaluation of the introduction of this prevention mechanism. Through a deterministic compartmental model calibrated to the Catalan situation, with both administrative and publicly-available data, the implementation of PrEP is found to be cost-saving in 16 years and 4 months, for a 40-years horizon, freeing 93.8 million euros from the healthcare payer perspective by averting infections, but generating an extra burden of 12.75 million for the society as a whole through work absences and transport costs. In terms of health indicators, 10,322 additional discounted QALYs are generated by the intervention. Global health results (discounted QALYs) are sensitive to sexual behavioral patterns among MSM, the discount rate and the utility perceived by people on antiretroviral therapy. The time needed to attain savings in costs and its magnitude are largely dependent on the price of PrEP (reduced if offered on-demand), its effectiveness, the discount rate and sexual behaviour.

## Supporting information

S1 Fig Deterministic compartmental mathematical model of HIV transmission. Source: Nichols et al. (2016) Supplementary Material. *: μ = mortality general population, μk = mortality untreated HIV-infected in infection stage k, μRx = mortality ARV treated, λ = force of infection, λP = force of infection while on PrEP.
(TIFF)

S2 Fig Differential equations. Where SP and Si for i = 1,2 stand for the number of susceptible individuals with and without PrEP, IPk and IIk for k = 1,…,9 stand for the number of infected, undiagnosed individuals with and without PrEP, and IRX stands for the number of treated individuals.
(TIFF)

S3 Fig Share of costs per group of items over time for PrEP scenario. Source: Own elaboration.
(TIFF)

S1 Table PrEP eligibility criteria in Spain. Source: Spanish Ministry of Health.
(TIFF)

S2 Table Description and calibration of the monthly baseline parameters of the model. (TIFF)

S3 Table Compartmental utility weights. *: No significant side effects reported from PrEP. Source: Cited individually.
(TIFF)

S4 Table Yearly PrEP costs for the initial year, in 2021 euros as financed by the SCS. Source: HUGTIP hospital pharmacy and Pep Coll, personal communication.
(TIFF)

S5 Table Yearly PrEP costs for t>12, in 2021 euros as financed by the SCS. Source: HUGTIP hospital pharmacy and Pep Coll, personal communication.
(TIFF)

S6 Table Yearly ARV costs for the initial year, in 2021 euros as financed by the SCS. Source: HUGTIP hospital pharmacy and Pep Coll, personal communication.
(TIFF)

S7 Table Yearly ARV costs for t>12, in 2021 euros as financed by the SCS. Source: HUGTIP hospital pharmacy and Pep Coll, personal communication.
(TIFF)
