# Supplementary figures and images for: Cost-effectiveness analysis of the daily HIV pre-exposure prophylaxis in men who have sex with men in Barcelona
Source: PLoS One. 2023 Jan 17;18(1):e0277571. doi: 10.1371/journal.pone.0277571 (PMC9844874; doi:10.1371/journal.pone.0277571)

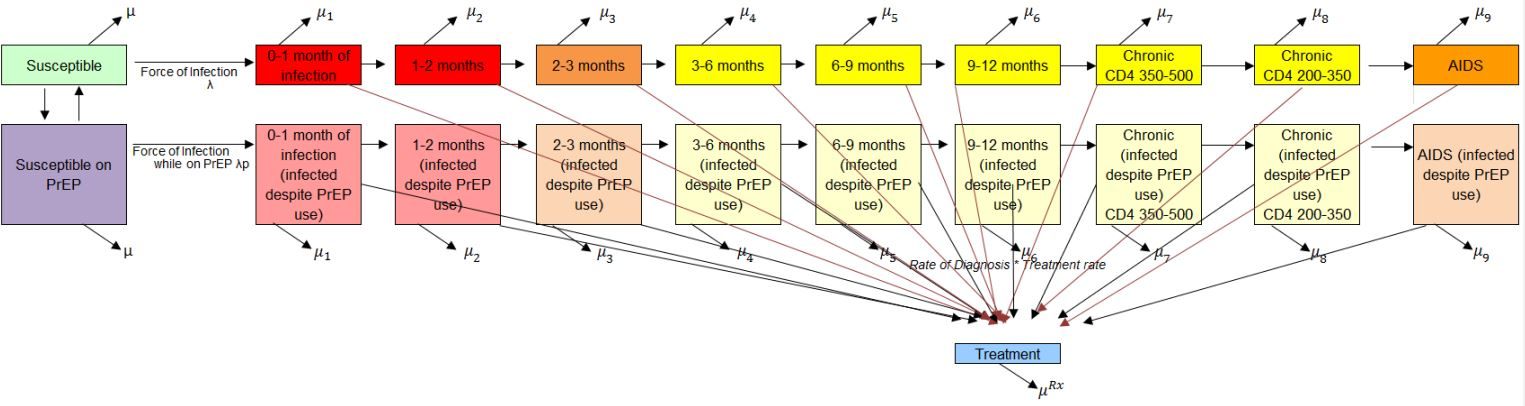

Supplement: S1 Fig — Source: Nichols et al. (2016) Supplementary Material. *: μ = mortality general population, μk = mortality untreated HIV-infected in infection stage k, μRx = mortality ARV treated, λ = force of infection, λP = force of infection while on PrEP. (TIFF) [file pone.0277571.s001.tiff]

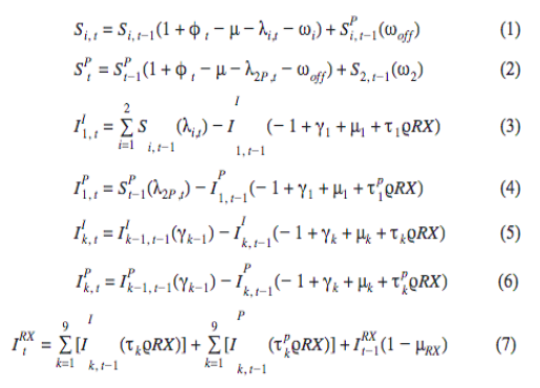

Supplement: S2 Fig — Where SP and Si for i = 1,2 stand for the number of susceptible individuals with and without PrEP, IPk and IIk for k = 1,…,9 stand for the number of infected, undiagnosed individuals with and without PrEP, and IRX stands for the number of treated individuals. (TIFF) [file pone.0277571.s002.tiff]

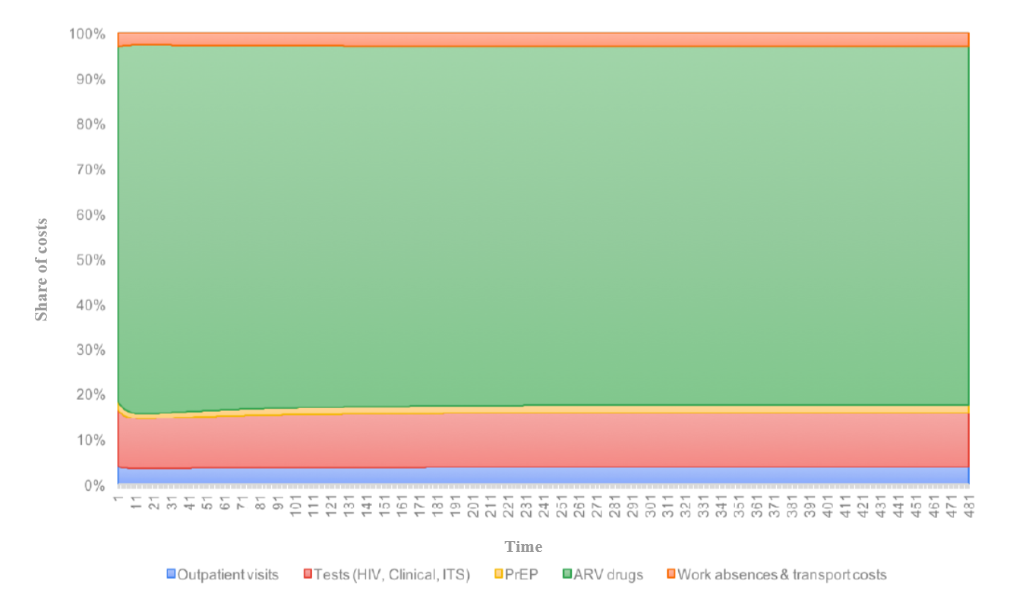

Supplement: S3 Fig — Source: Own elaboration. (TIFF) [file pone.0277571.s003.tiff]
